# HER2-Low Expression in Male Breast Cancer: Results from a Multicenter Series in Italy

**DOI:** 10.3390/cancers16030548

**Published:** 2024-01-27

**Authors:** Valentina Silvestri, Virginia Valentini, Agostino Bucalo, Giulia Conti, Livia Manzella, Daniela Turchetti, Antonio Russo, Carlo Capalbo, Laura Ottini

**Affiliations:** 1 Department of Molecular Medicine, Sapienza University of Rome, 00161 Rome, Italy; valentina.silvestri@uniroma1.it (V.S.); virginia.valentini@uniroma1.it (V.V.); agostino.bucalo@uniroma1.it (A.B.); giulia.conti@uniroma1.it (G.C.); carlo.capalbo@uniroma1.it (C.C.); 2 Department of Clinical and Experimental Medicine, University of Catania, 95123 Catania, Italy; manzella@unict.it; 3 Department of Medical and Surgical Sciences (DIMEC), University of Bologna, 40126 Bologna, Italy; daniela.turchetti@unibo.it; 4 Section of Medical Oncology, Department of Surgical and Oncological Sciences, University of Palermo, 90127 Palermo, Italy; antonio.russo@usa.net; 5 Medical Oncology Unit, Sant’Andrea University Hospital, 00189 Rome, Italy

**Keywords:** male breast cancer, HER2, HER2-low, *BRCA1/2*, trastuzumab deruxtecan, breast cancer

## Abstract

**Simple Summary:**

In the evolving landscape of breast cancer care, recognizing HER2-low expression as a target for anti-HER2 therapies has revolutionized treatment approaches. This retrospective, observational, multicenter study, focusing on male breast cancer (MBC), aimed to characterize the HER2-low subtype. Of the 144 MBCs analyzed, 54.9% were HER2-0, 27.1% HER2-low, and 18% HER2-positive. Specifically, among hormone receptor-positive MBCs, 34.8% were HER2-low. Notably, the prevalence of HER2-low in MBC appears slightly lower than in females. HER2-low MBCs showed a higher likelihood of positive lymph node involvement. About 13% of HER2-low MBCs carried germline *BRCA1/2* pathogenic variants, predominantly in *BRCA2*. Overall, our data derived from the largest MBC series to our knowledge, also characterized for germline pathogenic variants, support the recognition of the emerging category of HER2-low breast cancer. This study emphasizes the need for tailored understanding of HER2-low expression in MBC and underscores potential implications for refining therapeutic approaches based on distinct molecular profiles.

**Abstract:**

In the field of breast cancer care, a significant breakthrough has occurred with the recognition of HER2-low expression as a target for novel anti-HER2 antibody–drug conjugates (ADC). This discovery is reshaping the treatment landscape, challenging previous perceptions that considered HER2-low as clinically insignificant. The ability to target HER2-low expression is expected to have substantial clinical implications, irrespective of gender, including in cases of male breast cancer (MBC). However, an estimate of the prevalence of the HER2-low subtype in MBC is missing. This retrospective, observational, multicenter study was aimed at characterizing the HER2-low subtype in MBC. For the purpose of this study, the three-tiered categorization of HER2 (HER2-0, HER2-low, and HER2-positive) was used to reclassify the HER2-negative group into HER-0 or HER2-low subtypes. In the whole series of 144 invasive MBCs, 79 (54.9%) were HER2-0 (IHC scores of 0), 39 (27.1%) HER2-low (IHC scores of 1+/2+ with negative ISH), and 26 (18.0%) HER2-positive (IHC scores of 3+/2+ with positive ISH). Specifically, among hormone receptor-positive (HR+) HER2-negative invasive MBCs, 34.8% were HER2-low and 65.2% HER2-0. Compared with HER2-0, HER2-low subtype was associated with a positive lymph node involvement (*p* = 0.01). Other pathologic characteristics including histology, staging, and grading did not show notable variations between the two subtypes. The presence of germline *BRCA1/2* pathogenic variants (PVs) did not significantly differ between HER2-0 and HER2-low MBCs. However, about 13% of HER2-low MBCs had germline PVs in *BRCA1/2* genes, mainly *BRCA2*, a clinically relevant observation in the context of combined target therapy. Overall, our data, which focused on the largest gender-specific breast cancer series, to our knowledge, confirm that the emerging three-tiered categorization of HER2 (HER2-0, HER2-low, and HER2-positive) can also be considered in MBC, to mitigate both the gender gap and the underrepresentation of males in clinical trials.

## 1. Introduction

Breast cancer is a highly heterogeneous disease. Using gene expression profiles or immunophenotypic characteristics, different molecular subtypes of breast cancer have been identified, thus allowing for a refined treatment of breast cancer patients [1].

In clinical practice, routine immunohistochemistry (IHC) evaluation of well-established prognostic and predictive molecular biomarkers offers valuable information for clinical management, serving as a cost-effective alternative to genotyping assays. In particular, breast cancer molecular subtypes are simplified into three categories based on the IHC evaluation of three key biomarkers: estrogen receptor (ER) expression, progesterone receptor (PR) expression, and HER2 overexpression. These biomarkers serve both prognostic and predictive purposes, encompassing the biology of tumors and identifying three distinct entities: luminal-like, HER2-positive, and triple-negative tumors [1].

In the evolving landscape of breast cancer care, recognizing HER2-low expression as a target for anti-HER2 therapies has revolutionized treatment approaches. Among tumors conventionally defined HER2-negative, around half exhibit low levels of HER2 (HER2 1+ or 2+ with negative in situ hybridization (ISH)) and are currently categorized as HER2-low. HER2-low neoplasms more commonly express hormone receptors (HR+), but they are biologically and clinically diverse and tend to undergo changes in HER2 expression levels throughout the natural progression of the disease [2,3]. Current evidence is inconsistent to draw definitive conclusions regarding the prognostic role of HER2-low. Findings remain contentious across various studies, and the majority of evidence indicates no substantial influence on survival outcomes [4].

Recently, the definition of HER2-low status has gained significance as it predicts the response to novel anti-HER2 antibody-drug conjugates (ADC) such as trastuzumab deruxtecan (T-DXd). The advent of anti-HER2 ADC introduced a new therapeutic landscape for breast cancer patients, including those with low HER2 expression. T-DXd is an ADC that combines an anti-HER2 antibody (trastuzumab) with a potent cytotoxic payload. This drug has a unique feature in that it can effectively target tumor cells expressing low levels of HER2. In the DESTINY-Breast04 trial, T-DXd demonstrated superior efficacy compared to standard chemotherapy options in patients with HER2-low advanced breast cancer. Despite concerns about the accuracy of HER2-low scoring, it is noteworthy that a progression-free survival benefit was observed in patients with IHC 1+ and IHC 2+, ISH-negative disease. This suggests that conventional IHC tests can reliably identify patients who may benefit from T-DXd and emphasizes the clinical significance of the HER2-low patient population [2]. It is important to note that in clinical studies utilizing this new compound, males are either absent or represent a percentage lower than 1% [5,6,7,8].

Compared with female breast cancer (FBC), male breast cancer (MBC) is a rare and understudied disease. It represents less than 1% of all male tumors and about 1% of all breast cancers [9,10]. 

In Italy, about 600 men are estimated to be diagnosed with BC annually, and the standardized incidence rate of MBC is 1.7 × 100,000, with an increasing trend of incidence being observed [11]. As in FBC, HER2 is also an established clinical biomarker in MBC [12,13,14,15,16]. Notably, male breast tumors are more frequently HER2-negative compared with their female counterparts [12,16,17], thus highlighting the relevance of discriminating the HER2-low subtype in this population, in light of the novel clinical findings. Currently, there are no specific nor well-established data concerning HER2-low breast cancers arising in male population. Despite recent studies examining the impact of HER2-low expression in FBC series, the question regarding the incidence of HER2-low and whether it should be defined as a new subtype of MBC remains unresolved.

The aim of this study was to identify and characterize the emerging HER2-low subtype of breast cancer in a male-specific context.

## 2. Materials and Methods

This was an observational, retrospective study, based on a series of 144 invasive MBC cases collected from five Italian Research Centers participating in the Italian multicenter study on MBC [18]. All patients enrolled had been previously tested for germline pathogenic variants (PVs) in 50 breast cancer predisposition genes, including *BRCA1* and *BRCA2* [18,19].

The data collection involved gathering baseline pathological characteristics of patients, including tumor histotype, pathological TNM stage, lymph node involvement, grading, ER/PR status, Ki-67 index and HER2 status.

ER, PR, Ki-67, and HER2 status of breast tumors were obtained from IHC analysis of sections from formalin-fixed, paraffin-embedded primary mammary tumor blocks, performed at the center of enrollment. Consistency checks were performed to validate IHC data against supplementary scoring information provided. Central pathology review was not performed.

Specifically, ER/PR status was considered positive if positive nuclei were ≥1%; Ki67/MIB1 expression was considered high if positive nuclei were ≥20% [20,21].

Regarding HER2, MBCs were initially classified using the two-tiered HER2 (HER2-negative and HER2-positive) classification, according to an algorithm adapted from the 2018 American Society of Clinical Oncology–College of American Pathologists testing guidelines [22]. For the purpose of this study, the three-tiered categorization of HER2 (HER2-0, HER2-low, and HER2-positive), was used to reclassify the HER2-negative group into HER-0 or HER2-low, according to the recently proposed HER2-low scoring algorithm from the DESTINY-Breast04 trial [2]. In particular, MBC cases with IHC score of 0 were classified as HER2-0, while cases with IHC scores of 1+ or 2+ and negative ISH were classified as HER2-low.

Categorical variables underwent analysis through the chi-square test or Fisher’s exact test. *p*-values ≤ 0.05 were considered statistically significant. Statistical tests were employed to examine the significance of associations and differences in the data, employing these methods to ensure a comprehensive evaluation of the study parameters.

All patients gave informed consent for the use of tissue for research purposes and biomarker evaluation; this was approved by the Local Ethical Committee (Sapienza University of Rome, Protocol 669/17) and was performed according to Helsinki’s declaration.

## 3. Results

**Figure 1:**
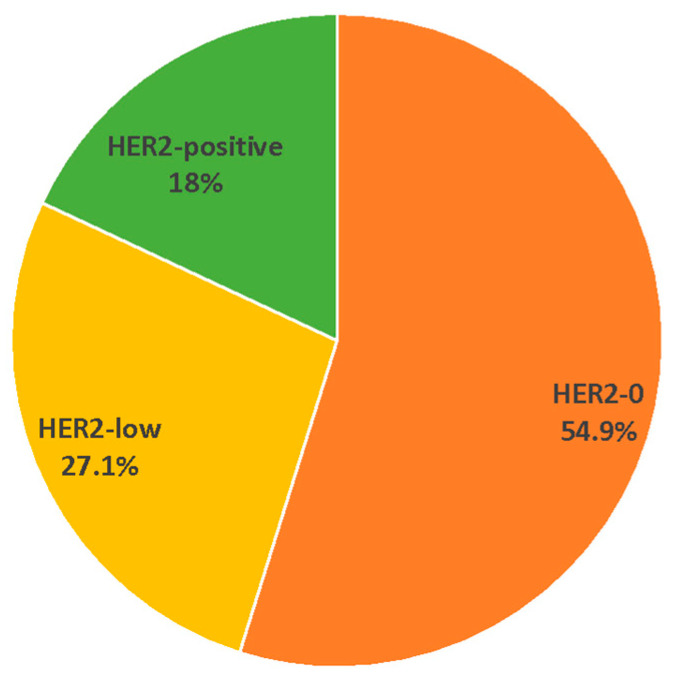
Percentage of HER2 subtypes in the whole series of 144 invasive MBCs.

In this comprehensive study involving 144 invasive male breast tumors, the emerging three-tiered categorization of HER2 (HER2-0, HER2-low, and HER2-positive) was applied. MBC cases were classified as HER2-0 with IHC scores of 0, as HER2-low with IHC scores of 1+ or 2+ and negative ISH, and as HER2-positive with IHC scores of 3+ or 2+ and positive ISH. Overall, 79 tumors (54.9%) were classified as HER2-0, 39 (27.1%) as HER2-low, and 26 (18.0%) as HER2-positive (Figure 1).

Three MBCs (2.1%) were triple-negative, and four (2.8%) were either ER- or PR-negative. As the subsequent analyses were focused on HR+ MBCs, these tumors were excluded. Notably, no HER2-low male breast tumor was found among the seven excluded samples. Among the 112 HR+ MBCs classified as HER2-negative, 73 cases (65.2%) fell under the HER2-0 category, while 39 cases (34.8%) were classified as HER2-low (Table 1).

Ductal histological type was predominant in both subtypes, with 93.2% in HER2-0 and 92.3% in HER2-low, yielding a non-significant difference. Staging comparisons showed no significant variations between the two subtypes for both Stage I and Stage ≥ II. However, lymph node involvement displayed a significant difference (*p* = 0.01), with 74% of HER2-low cases having lymph node-positive status compared to 55% in HER2-0. Grading analysis showed a balanced distribution between Grade 1 and Grade ≥2 in both subtypes, with no significant difference observed. Similarly, the Ki67 proliferation index did not exhibit statistically significant differences. In the series analyzed, 18 MBCs were carriers of germline predisposing PVs in *BRCA1/2* genes, 17 in *BRCA2* and 1 in *BRCA1*. The presence or absence of *BRCA1/2* PVs did not significantly differ between the subtypes. The single *BRCA1* PV was identified in a HER2-low MBC. Notably, the only *CHEK2* PV detected in the entire series was identified in a HER2-low MBC. 

## 4. Discussion

As clinical practices continue to advance, there is an imperative for a comprehensive molecular characterization of patient subgroups that goes beyond the conventional binary classification of HER2 status. Notably, approximately half of female patients with HER2-negative breast cancer exhibit HER2-low expression status. This nuanced understanding becomes particularly significant in the realm of MBC, providing insights into potential variations in response to novel drugs and laying the groundwork for more refined management strategies in the future [4]. Despite numerous recent efforts to delineate the characteristics of HER2-low breast cancers, there remains a noticeable gap in the literature concerning reliable HER2-low diagnoses in MBC. In this context, we undertook the evaluation of HER2-low tumors within the MBC population.

Our study provided a refined distribution of HER2 subtypes among male breast tumors, showing that about 55% were HER2-0, 27% HER2-low, and 18% HER2-positive MBCs. The percentage of HER2-low appears to be lower compared to what is reported in female cases, where the presence of HER2-low accounts for approximately 45–55% of all breast cancer cases [2]. In the data available from unselected populations and national registries, the percentage of HER2-low breast cancers in the male population is extremely variable, ranging from approximately 40% to about 70% [5,6,7,8,23]. In the study conducted at the mono-institutional Samsung Medical Center, which included 51 males out of a total of 10,186 patients, as well as in two recent studies utilizing nationwide data from the Korean Breast Cancer Registry (122 males out of 23,539 patients) and the Austrian AGMT_MBC-Registry (17 males out of 1973 patients), no notable gender-related differences appear to have been identified. It is essential to underscore, however, that the Austrian AGMT_MBC-Registry and the study by Park et al. involved significantly smaller sample sizes (approximately 17 and 51 male patients, respectively) in comparison to our study [5,6,7,8]. Additionally, the extended enrollment period of the Korean study, coupled with variations in the interpretation of HER2 expression levels, may have impacted the ability to discern significant differences between HER2 subtypes.

Overall, to the best of our knowledge, the current study appears to be the first one focused exclusively on the male population with HER2-0/HER2-low and with the largest sample size reported in specific medical literature. Two previous studies, analyzing HER2 by IHC and ISH in MBC cases, provided detailed information allowing for an extrapolation of HER2-low status [24,25]. Data from these studies appeared to confirm a percentage around 25% of HER2-low in MBC.

In FBC, the HER2-low subtype was observed more frequently in HR+ tumors [26,27]. In our MBC series, HER2-low was observed in HR+ tumors only, probably due to the rarity of the HR-negative subtype in MBC.

Although, in our data, the percentage of HER2-low tumors is slightly lower compared to the female counterpart, it still remains of considerable magnitude, with approximately 35% of all MBCs classified as HER2-negative by standard analyses. This not only accentuates the need for a tailored understanding of HER2-low expression in MBC but also emphasizes the potential for refining therapeutic approaches based on these distinctive molecular profiles.

In the recently reported studies, diverse conflicting data regarding differences in various histopathological characteristics between the two HER2-0 and HER2-low subgroups do not allow for clear associations to be identified. Here, we characterized HR+, invasive MBCs according to HER2-0 and HER2-low status, in order to identify possible distinctive pathologic features able to discriminate between the two subtypes and provide useful clinical information.

In our study, HER2-low MBCs displayed a positive lymph node involvement more frequently than HER2-0 MBCs. This result may suggest that HER2-low BCs might be more similar to HER2-positive than to HER2-negative tumors, displaying pathology characteristics suggestive of a more aggressive phenotype. Other parameters such as histological type, staging, grading, Ki67 proliferation index, and the presence of germline *BRCA1/2* PVs did not exhibit significant variations between the two subtypes. Our data on histopathological characteristics are quite consistent with the existing literature [28,29,30], although currently, it is not possible to definitively confirm the association of HER2-low with other classic histopathological features indicating a poor prognosis.

Interestingly, in our study, about 13% of HER2-low MBC cases showed predisposing PVs in *BRCA1/2* genes, the great majority in *BRCA2*. Conversely, in the gender-unselected population, less than 2% of HER2-low breast tumors seemed to carry PVs in *BRCA1/2* genes, and PVs in these genes were significantly more frequently identified in HER2-low patients [3]. Our data suggested that the male HER2-low category appears to be enriched with mutated *BRCA2* tumors compared to the female counterpart. This may be possibly due to the well-known gene- and gender-specific role of *BRCA1/2* PVs in the two sexes, with *BRCA1* PVs mainly involved in females, while *BRCA2* PVs are mainly involved in male breast cancers. It is noteworthy that compared with *BRCA2*-associated FBCs, *BRCA2*-associated MBCs were more likely to be HR+ and HER2-negative [31]. Thus, it may suggest that among *BRCA2*-associated male breast tumors classified as HER2-negative, there may be a relevant proportion of HER2-low.

Moreover, transcriptomic data on MBC showed that male breast tumors associated with germline *BRCA1/2* PVs and those with a high immune involvement were also characterized by a high HER2 signaling score, despite being classified as HER2-negative by IHC, suggesting that the HER2 pathway may be active [32,33,34]; thus, they might benefit from treatment with T-DXd, or from a combined therapeutic approach including PARPi and/or immunotherapy.

This study has some limitations. Firstly, it is a retrospective, multicenter study, which may introduce variability in data collection and interpretation. Furthermore, the lack of a central pathology review could affect the consistency of HER2 status classification. However, data collected are representative of the typical pathology assessment conducted in routine practice in our country, and the distribution of HER2 status across different study centers was generally consistent. Moreover, as a retrospective study, it lacks longitudinal follow-up data, which could provide insights into the long-term clinical outcomes of HER2-low MBC patients.

## 5. Conclusions

Overall, our data from a large case series of male breast carcinomas in the Italian population confirm that the emerging three-tiered categorization of HER2 (HER2-0, HER2-low, and HER2-positive) can also be considered in MBC. Further research and prospective studies are warranted to validate these observations and guide tailored treatment strategies [35]. Nonetheless, it is crucial to highlight that in clinical studies employing novel compounds, MBC patients are absent, or their representation is lower than 1%. This underrepresentation raises concerns about the generalizability of findings and the application of treatment outcomes to male patients. Inclusion of a more substantial number of male cases in future clinical studies is crucial to ensure a comprehensive understanding of the drug’s efficacy, safety, and potential gender-specific responses. Moreover, it will contribute to refining treatment strategies and tailoring therapies for male patients with breast cancer, fostering more equitable and effective healthcare practices.

## Figures and Tables

**Table 1:** Pathologic characteristics according to HER2-0 and HER2-low status in HR+ invasive MBCs.

Characteristics ^1^		HER2-0 (N = 73)	HER2-Low (N = 39)	*p*-Value ^2^
Histological type	Ductal	68 (93.2%)	36 (92.3%)	0.87
	Other	5 (6.8%)	3 (7.7%)	
Stage	I	15 (26.8%)	11 (37.9%)	0.29
	≥II	41 (73.2%)	18 (62.1%)	
Nodes	Negative	33 (45%)	7 (26%)	0.01
	Positive	27 (55%)	20 (74%)	
Grading	1	3 (4.4%)	3 (8.1%)	0.44
	≥2	65 (95.6%)	34 (91.9%)	
Ki67	Low	24 (34.8%)	13 (35.1%)	0.97
	High	45 (65.2%)	24 (64.9%)	
Germline *BRCA1/2* PVs	Negative	60 (82.2%)	34 (87.2%)	0.49
	Positive	13 (17.8%)	5 (12.8%)	

^1^ Some data for each pathologic characteristic are not available. ^2^ *p*-values from chi-square test or Fisher’s exact test.

## Data Availability

Data are contained within the article.
